# Loss in lung volume and changes in the immune response demonstrate disease progression in African green monkeys infected by small-particle aerosol and intratracheal exposure to Nipah virus

**DOI:** 10.1371/journal.pntd.0005532

**Published:** 2017-04-07

**Authors:** Yu Cong, Margaret R. Lentz, Abigail Lara, Isis Alexander, Christopher Bartos, J. Kyle Bohannon, Dima Hammoud, Louis Huzella, Peter B. Jahrling, Krisztina Janosko, Catherine Jett, Erin Kollins, Matthew Lackemeyer, Daniel Mollura, Dan Ragland, Oscar Rojas, Jeffrey Solomon, Ziyue Xu, Vincent Munster, Michael R. Holbrook

**Affiliations:** 1 NIAID Integrated Research Facility, Ft. Detrick, Frederick, Maryland, United States of America; 2 Center for Infectious Disease Imaging, NIH Clinical Center, Bethesda, Maryland, United States of America; 3 Emerging Viral Pathogen Section, NIAID, Ft. Detrick, Frederick, Maryland, United States of America; 4 Clinical Research Directorate/Clinical Monitoring Research Program, Leidos Biomedical Research, NCI Campus, Frederick, Maryland, United States of America; 5 Virus Ecology Unit, Laboratory of Virology, Rocky Mountain Laboratories, NIAID, Hamilton, Montana, United States of America; University of Oxford, UNITED KINGDOM

## Abstract

Nipah virus (NiV) is a paramyxovirus (genus *Henipavirus*) that emerged in the late 1990s in Malaysia and has since been identified as the cause of sporadic outbreaks of severe febrile disease in Bangladesh and India. NiV infection is frequently associated with severe respiratory or neurological disease in infected humans with transmission to humans through inhalation, contact or consumption of NiV contaminated foods. In the work presented here, the development of disease was investigated in the African Green Monkey (AGM) model following intratracheal (IT) and, for the first time, small-particle aerosol administration of NiV. This study utilized computed tomography (CT) and magnetic resonance imaging (MRI) to temporally assess disease progression. The host immune response and changes in immune cell populations over the course of disease were also evaluated. This study found that IT and small-particle administration of NiV caused similar disease progression, but that IT inoculation induced significant congestion in the lungs while disease following small-particle aerosol inoculation was largely confined to the lower respiratory tract. Quantitative assessment of changes in lung volume found up to a 45% loss in IT inoculated animals. None of the subjects in this study developed overt neurological disease, a finding that was supported by MRI analysis. The development of neutralizing antibodies was not apparent over the 8–10 day course of disease, but changes in cytokine response in all animals and activated CD8+ T cell numbers suggest the onset of cell-mediated immunity. These studies demonstrate that IT and small-particle aerosol infection with NiV in the AGM model leads to a severe respiratory disease devoid of neurological indications. This work also suggests that extending the disease course or minimizing the impact of the respiratory component is critical to developing a model that has a neurological component and more accurately reflects the human condition.

## Author summary

Nipah virus (NiV) was identified in the late 1990s as the causative agent of severe respiratory and neurological disease in Malaysia and Bangladesh. The virus is transmitted by inhalation, contact or consumption of contaminated material. In this study, our objective was to characterize NiV-induced disease progression in the African Green Monkey model utilizing clinical imaging capabilities. In this work, we also provide the first temporal evaluation of the immune response to infection following NiV infection and the first characterization of disease following aerosol exposure. Here, we found that NiV infection following intratracheal and aerosol exposure lead to a severe respiratory disease and rapid disease course with no overt clinical evidence of neurological disease. Despite the rapid course of disease, changes in the cytokine response and peripheral immune cell populations suggest development of a cell-mediated immune response in the latter stage of disease. While the current model for evaluating NiV infection is useful for testing of medical countermeasures, further work is required to understand how this model can represent human disease.

## Introduction

Nipah virus (NiV) (Family *Paramyxovirus*, genus *Henipavirus*) emerged in Malaysia in the late 1990s where it caused a significant outbreak of respiratory and neurological disease in people working closely with pigs in both Malaysia and Singapore [1]. This outbreak resulted in the death of at least 105 humans and the culling of more than one million pigs [1]. In 2001, NiV re-emerged in Bangladesh [2] and has become a regular cause of severe disease in parts of rural Bangladesh and India where over 240 people have died from the infection [3]. Transmission of NiV to human populations in Bangladesh and India results from contact with the carcasses or excreta of *Pteropus spp*. bats, also known as flying foxes. In Bangladesh, there is a strong correlation in human cases of NiV infection with consumption of unpasteurized, and possibly fermented, date palm sap containing bat excreta [4–6]. In addition to transmission from bats and pigs, there has also been evidence of human-to-human transmission, including nosocomial infections [7, 8].

NiV infection causes an acute febrile disease with rapid onset that is typically characterized by areflexia, seizures, muscle spasms, hypertension, cough, vomiting and development of atypical pneumonia and severe respiratory disease [3, 9]. In a number of cases, development of neurological symptoms has been documented with instances of neurological relapse or late onset encephalitis in 5–10% of the cases [10], with occurrence up to 11 years after the initial virus exposure [11]. NiV infection has a case fatality rate of around 54% with fatality rates ranging from 40–100%, depending upon the outbreak [12]. NiV infection in humans takes the form of an acute respiratory disease (ARD) or atypical pneumonia with development of neurological disease, or, in some cases, only a neurological disease [3, 9, 11]. Many survivors of NiV infection develop long-term neurological sequelae including myoclonus, cognitive dysfunction, personality changes and persistent abnormalities on brain magnetic resonance imaging (MRI) examinations [13, 14]. The use of x-ray imaging in a Bangladesh outbreak described bilateral “ground glass” opacities indicative of acute respiratory distress syndrome [15]. In the case of neurological disease, MRI identified focal hyperintensities in the white matter of the parietal lobes of one patient from Malaysia that may have been evidence of microinfarctions [11, 16, 17]. Some of these lesions cleared over time, while others persisted [18]. Other studies of neurological disease in humans have described confluent cortical involvement rather than discrete lesions [19].

Previous work has identified the African green monkey (AGM) as a model for NiV-induced disease. Infection of AGM by intratracheal (IT) inoculation results in a largely lethal disease with evidence of respiratory infection including enlarged lungs, hemorrhage and consolidation and blood in the pleural fluid [20–22]. Congestion within the meninges, meningeal hemorrhage and edema in the brain has also been reported [23]. Histological evaluation identified systemic vasculitis and evidence of syncytia in the spleen, kidney and pancreas [20]. Intraperitoneal inoculation of NiV in AGM resulted in similar disease with pulmonary consolidations and evidence of developing encephalitis [24].

The objective of the studies described here was to determine if small particle aerosol infection resulted in a markedly different disease than IT inoculation. In order to address this objective we utilized computed tomography (CT) and MRI to characterize and quantify disease progression. We also evaluated changes in peripheral and infiltrating immune cell populations in order to determine if immunopathogenesis was a critical component of the disease process. In these studies we found that IT inoculation induced largely focal consolidation within the lung with a rapid decrease in lung volume, while aerosol inoculation caused a more diffuse change, but with similar outcome. Gross observation of animals indicated that all succumbed to an acute respiratory disease, a finding that was supported by CT. MRI found evidence of venous abnormalities in the brain of only two animals. Histological evaluation of tissues identified widespread vasculitis, as previously described, in both aerosol and IT exposed animals. Evaluation of the immune response provided evidence of a systemic inflammatory response that was highlighted in lung lesions where there was a significant increase in pro-inflammatory cytokines. Changes in peripheral T cell populations also indicated expansion of CD8+ T cells, but little change in CD4+ T cells over the 8–10 day course of infection. These data demonstrate that small particle aerosol inoculation of NiV causes a disease that is more broadly disseminated in the lung than IT inoculation, but that is equally lethal in the AGM model with a rapid inflammatory response and pulmonary infiltration with minimal evidence of neurological disease.

## Materials and methods

### Ethics statement

The Nipah-Malaysia virus that was used in this study was isolated from a human case in 1996 and initially provided to USAMRIID from an existing collection by the Special Pathogens Branch, Centers for Disease Control, Atlanta, GA. The IRF received the virus from the USAMRIID collection.

Work with non-human primates was conducted in accordance with an Animal Study Protocol approved by the NIAID Division of Clinical Research Animal Care and Use Committee (Protocol #IRF-034E) following recommendations in the Guide for the Care and Use of Laboratory Animals. This institution also accepts as mandatory the Public Health Service policy on Humane Care and Use of Laboratory Animals. All animal work at NIAID was performed in a facility accredited by the Association for the Assessment and Accreditation of Laboratory Animal Care International (AAALACI).

Non-human primates were housed either singly or in pairs in an ABSL-2 facility prior to introduction into the BSL-4 facility. Animals were singly housed during BSL-4 acclimatization and during the course of the study. At all times animals were provided with appropriate enrichment including, but not limited to, polished steel mirrors and durable toys. Animals were anesthetized in accordance with BSL-4 standard protocols prior to all procedures including inoculation, imaging and collection of blood to minimize stress to the animals. Animals were observed following anesthesia to ensure complete recovery. All work with non-human primates was done in accordance with the recommendations of the Weatherall Report.

### Virus and cells

These studies utilized the Malaysian strain of Nipah virus (NiV). The challenge stock was cultivated in VeroE6 cells following receipt from USAMRIID. USAMRIID passage history included three passages in VeroE6 cells and one passage in Vero cells followed by two subsequent passages in our facility. VeroE6 cells (BEI #NR596) were maintained in α-MEM w/GlutaMAX and incubated at 37°C/5% CO_2_. All work with viable NiV was performed in the BSL-4 facility at the NIAID Integrated Research Facility in Frederick, MD.

### Animals

Wild-caught Caribbean origin African green monkeys (AGM) were purchased from PrimGen (Hines, IL). Animals were group housed prior to being assigned to study and singly housed during the course of the study. Two males and one female were included in each study group. Animals were identified for inclusion based on similarity of size. All work in this study was reviewed and approved by the NIAID Division of Clinical Research (DCR) Animal Care and Use Committee (ACUC) (see Ethics Statement below).

### Inoculation

Animals in the first group (n = 3) were each inoculated via the intratracheal (IT) route with a target dose of 1x10^4^ pfu in a total volume of 1.0 ml of virus diluted in serum-free α-MEM using a bronchoscope to deposit 0.5 ml in each mainstem bronchus. Back calculation of the inoculum indicated a dose of 1.3x10^4^ pfu was delivered to each animal.

The animals in the second group (n = 3) were exposed to a small particle aerosol challenge using a 16 Liter, head-only aerosol exposure chamber and an aerosol management platform (AeroMP, Biaera Technologies, USA) within a Class III biosafety cabinet (Germfree, FL, USA). The animals were anesthetized and received a single, time calculated aerosol challenge with a target dose of 1x10^4^ pfu. Respiration values for the AGM were obtained using Guyton’s formula, MV = 2.1(BW^0.75), with an anesthesia adjustment, where MV is minute volume and BW is the body weight in grams [25]. Aerosol particles were generated by a 3-jet Collison nebulizer (BGI by Mesa Labs, NJ, USA) operating at 7.5 LPM (25–30 PSIG), which produced small particles ranging from 0.5–3 μm in size targeting the lower respiratory tract and alveolar region. An aerodynamic particle-sizing device (Aerodynamic Particle Sizer, TSI, MN, USA) verified particle size with real-time measurements (1.5 μm Mass Median Aerodynamic Diameter; 2.1 Geometric Standard Deviation). SKC biosamplers (SKC Inc., PA, USA) containing Dulbecco’s Modified Eagle Medium (Lonza, MD, USA), bovine serum albumin (Sigma-Aldrich, MO, USA), and antifoam SE-15 (Sigma-Aldrich, MO, USA) operated at a continuous flow rate of 13.0 LPM and collected aerosol challenge material to determine the aerosol concentration within the exposure chamber. An air wash period of 5 minutes between each challenge allowed the particles within the exposure chamber to decay [26]. A presented dose was calculated using the simplified formula D = R x C_exp_ x T_exp_, where D is the presented or estimated inhaled dose (PFU), R is the respiratory minute volume (L/min), C_exp_ is the aerosol concentration (PFU/L), and T_exp_ is the duration of the exposure (min). These formulae have been outlined previously [27]. The three animals received an average presented dose of 4.03x10^4^ pfu NiV. The dose per animal ranged from 3.04x10^4^ pfu to 4.06x10^4^ pfu based on back titration of the samples collected in biosamplers. One animal (#8095) had nasal discharge from one nostril during the aerosol exposure that potentially reduced the total dose delivered.

### Virus titration

Virus stocks and tissue samples were titrated in VeroE6 cells by plaque assay. Test samples were serially diluted 10-fold and inoculated onto multiwell plates containing nearly confluent VeroE6 cells. The virus was allowed to adhere and infect for 1h at 37°C/5% CO_2_. Following infection, cells were overlaid with semi-solid 1.25% Avicel (f/c) (FMC Biopolymer) diluted in EMEM. The cells were then incubated for 5 days at 37°C/5% CO_2_. Following incubation, the Avicel overlay was removed by aspiration and the cells were fixed and stained with neutral buffered formalin (NBF) containing 0.4% crystal violet for 30 min at room temperature. The plates were then washed with water and plaques enumerated.

### Imaging

Subjects were sedated and an intravenous catheter was placed in the cephalic vein prior to being intubated and taken to the imaging suite where they were immobilized using isoflurane, and positioned on the scanner bed in a supine fashion. All subjects underwent imaging on an Achieva 3 Tesla clinical MR scanner and a Precedence single photon emission computed tomography (SPECT)/CT unit (Philips Healthcare, Cleveland, OH, USA).

A brief, high resolution CT scan of the AGM torso was acquired in the trans-axial plane during a breath hold. CT parameters used for the acquisition included 140 kVp (kiloVolts to peak), 300 mAs (milliAmperes * second), and a slice thickness of 0.8 mm with a 0.40 mm increment, a matrix size of 512 x 512. Images were reconstructed to a 160 mm field of view resulting in a pixel size of 0.3 mm x 0.3 mm. To quantify CT data, lung regions were extracted in three steps: first, lung delineation was applied using a previously tested method [28]; then, each baseline lung mask was mapped to post-infection images via registration for refinement; and finally to further ensure the accuracy, the resulting lung segmentations were manually examined and adjusted.

Within each segmented lung region, volumetric quantifications were then calculated based on Hounsfield unit thresholds determined from a histogram based approach [29].

Brain MR images were obtained using a pediatric head/neck coil (head element selection only). During each imaging session, a series of sequences were obtained to examine the brain for indications of inflammation or lesions due to Nipah virus infection. These included a magnetization-prepared rapid gradient echo (MPRAGE) and a fluid attenuated inversion recovery (FLAIR) in addition to T_2_- weighted and T_1_-weighted fast field echo (FFE) sequences. Using a modification of an approach developed by Moonen et al., [30], alterations in vasculature were monitored using the principle of echo shifting (PRES) technique to produce a heavily weighted T2* image in order to examine changes in susceptibility contrast. The FLAIR and T_1_-weighted FFE were performed before and after injection with Magnevist (0.1 ml/kg) contrast agent to determine if there was disruption of the blood brain barrier. A pre-charged line with Magnevist was attached to the IV catheter and used for manual injection followed by a saline bolus to flush the system.

### Clinical analysis

Serum chemistries were run on an Abaxis Piccolo using the General Chemistry 13 standardized analysis panel (Abaxis). Complete blood counts (CBC) in whole blood were run with a five-part differential and reticulocytes on a Sysmex XT2000*i*V (Sysmex). All data were analyzed and graphed using Prism (GraphPad).

### Necropsy and histopathology

Complete necropsies were performed on each subject with tissues samples collected from the same region of individual tissues from each animal. Tissues collected for histopathology were fixed with 10% neutral buffered formalin (NBF) prior to removal from the biocontainment space. The tissues were embedded in paraffin, sectioned, mounted and stained with hematoxylin & eosin (H&E) prior to microscopic assessment. Brain and lung tissue samples collected for immune cell analysis (see below) were collected from the cerebral cortex and left caudal lobe, respectively.

### Immune cell analysis

PBMCs were isolated from whole blood using gradient purification within 4 hours of collection. Briefly, whole blood was overlain on Histopaque-1077 and then centrifuged (20 min, 800xg) with low brake. The cells at the interphase were collected and suspended in Hank’s Buffered Salt Solution (HBSS)+2% FBS. The cells were pelleted (10 min, 250xg) and re-suspended in HBSS+2% FBS prior to counting. Following isolation, PBMCs were aliquoted to flow tubes at 1x10^6^ cells per tube and then were stained with fluorophore-conjugated antibodies using two individual panels, one focused on T cells and the second on “other” cell populations with unstained controls in parallel. The T cell and “other” cell panels are described in S1 Fig. Cells in the T cell panel were re-suspended in PBS+2% FBS and incubated at 37°C for 1 hour prior to staining. All cells were then pelleted (5 min, 250-300xg) prior to adding the antibody cocktail. Cells were mixed and incubated for 20 min at room temperature in the dark. The cells were washed once with PBS+2% FBS and pelleted (5 min, 300xg) and the supernatant removed. The cells were fixed by addition of BD Cytofix/Cytoperm (BD Biosciences) and incubating cells in the dark for 30 min at room temperature. The cells were pelleted (1 min, 500xg) and re-suspended in Perm Wash (BD Biosciences) twice to wash the cells. The cells were re-suspended in Perm Wash prior to acquisition. Data were acquired on an LSR Fortessa (BD Biosciences) housed within the BSL-4 facility. All samples were compensated using the appropriate IgG1k or ArC reactive beads.

Cells from lung and brain collected at necropsy were isolated by collagenase digestion. Tissues were minced in a solution of collagenase D (100U/mL) and DNase I (100U/mL) and incubated at 37°C for 30 min. The digestion media was strained through a 100 μm cell strainer and collected. Residual tissue in the strainer was dissociated using the flat end of a syringe plunger and collected in the digestion media. Cells were pelleted (10 min, 280xg) and the supernatant removed. Lung cells were re-suspended in ACK Lyse (Quality Biologics) and incubated for 5 min at room temperature to lyse residual red blood cells. The cells were then pelleted (10 min, 280xg) and the supernatant discarded. Brain cell pellets were re-suspended in Percoll stock solution (1.13 g/L). The brain cell suspension was then underlain with Percoll working stock solution (1.008 g/L). Tubes were centrifuged (20 min, 1200xg) with minimal brake. The interface was collected, the cells pelleted (4 min, 1000xg) and the supernatant discarded. Cells were re-suspended in PBS and counted. The cells were then stained as described above with fluorophore-conjugated antibodies using two separate panels, one primarily focused on T and NK cells and the second on myeloid cells. The myeloid and TNK panels are described in S1 Fig. Data was collected on an LSR Fortessa (BD Biosciences) and analyzed with FlowJo analysis software. Gating strategies for these analyses are provided in S2–S5 Figs.

### Cytokine analysis

Cytokines from serum or supernatants from clarified tissue homogenates were tested using a standard 23-plex NHP cytokine assay panel (Millipore) and were stained following the manufacturer’s instructions. All samples were tested in duplicate wells. The panel was run on a FlexMap analysis system running xPonent software. Data was analyzed and graphed using Microsoft Excel.

### PCR analysis

PCR analysis was completed using an in-house assay that targets only the viral genomic RNA and not replication intermediates by using primers and probe that bind only in the intergenic region between the viral fusion and glycoprotein genes. The forward primer used for this assay is 5’-CCGTGAATATGTAATTGATAATTTCCC-3’ (Integrated DNA Technologies (IDT), Coralville, IA) and the reverse primer 5’-GCTTAGAAAGATACAGTTAAGTATCCAATGA-3’ (IDT). The probe is FAM-5’-CTTAGGACCCAGGTCCATAA-3’ Applied Biosystems, Inc. (ABI), Life Technologies, Grand Island NY). The assays were run on Trizol extracted RNA, isolated following manufacturer’s instructions, using either an ABI7500 or a Light Cycler real-time PCR instrument.

### Statistical analysis

Statistical analysis was not completed in this study. The small group sizes and out-bred nature of the animals used in this study limited the value of any statistical analyses. Where indicated, group means are presented and individual animals are identified to correlate specific findings with individual animals.

## Results

### NiV disease

**Fig 1:**
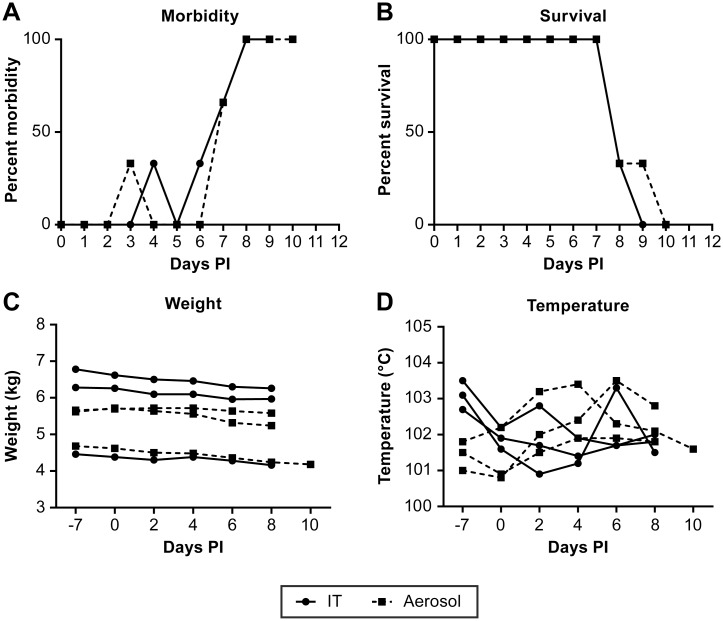
Clinical assessment of NiV infected AGMs. Assessment of animal morbidity (A), survival (B), weight (C) and body temperature (D) of the course of disease following NiV infection. IT inoculated animals are indicated by (●) and aerosol inoculated animals by (■). Morbidity was determined based on an animal “scoring” on a 10 –point clinical assessment scale based on cage-side observations.

Animals infected by either the IT or small particle aerosol route developed apparently similar diseases with the primary clinical manifestations including lethargy, cough, difficulty breathing and decreased fluid and food consumption. There were no overt clinical signs of neurological disease or hemorrhage and no indication of a “bloody froth” from the mouth as has been previously reported in this model [23]. Disease onset was clearly evident beginning on day 6 post infection (Fig 1A). The survival time for both IT and aerosol challenge groups was the same (~ 8 days) (Fig 1B), with the only animal surviving longer potentially having a reduced infection dose during aerosol exposure due to the presence of a nasal discharge from one nostril. There was no significant weight loss in any of the animals and only three animals had a body temperature that was moderately elevated over the course of disease (Fig 1C and 1D).

**Fig 2:**
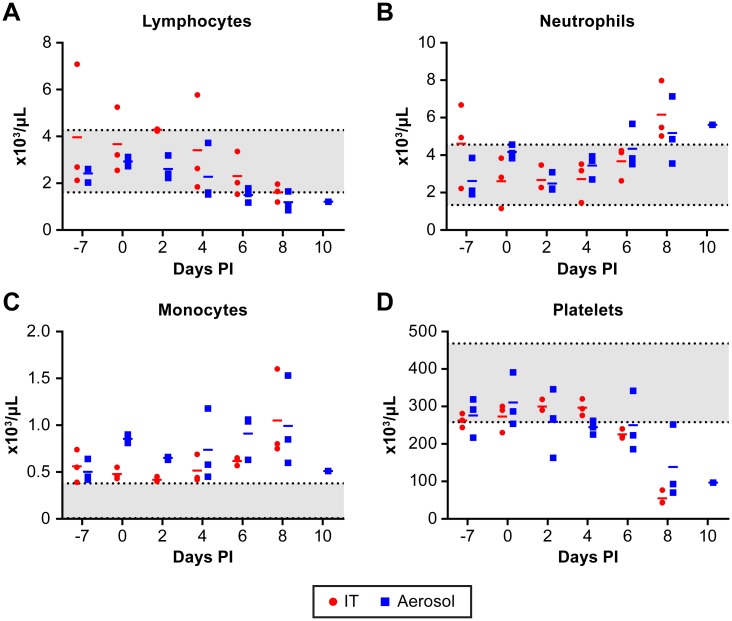
CBC on NiV infected AGMs. Temporal analysis of lymphocytes (A), neutrophils (B), monocytes (C) and platelets (D) demonstrating lymphopenia, neutrophilia and monocytosis developing over the course of disease. The rapid drop in platelet count occurs in the terminal phase of disease. IT inoculated animals are indicated by (●) and aerosol inoculated animals by (■). The mean for the group is indicated by a dash (-). The shaded area indicates the normal range for the specified value [44].

**Fig 3:**
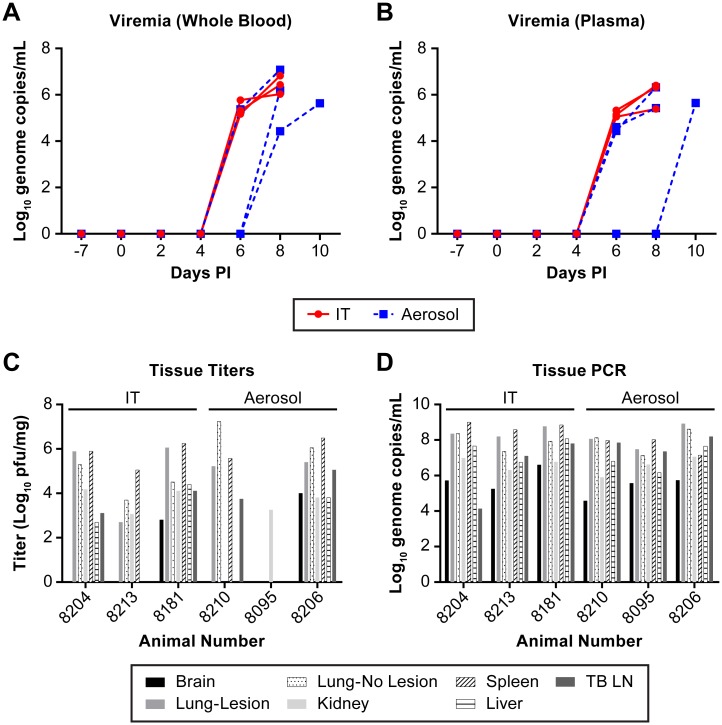
Distribution of viral RNA in blood and virus and viral RNA in tissues at necropsy. The presence of viral genomic RNA was determined by RT-PCR analysis in whole blood (A) and in plasma (B). The presence of virus in tissues was determined by plaque assay (C) and PCR for genomic RNA (D). In panels A and B, IT inoculated animals are indicated by (●) and aerosol inoculated animals by (■). Individual animal identifiers are indicated in panels C and D. These data demonstrate rapid amplification of virus in the blood and the broad distribution of NiV in AGM at necropsy.

Changes in blood chemistry were largely unremarkable while all animals developed lymphopenia, neutrophilia and monocytosis (Fig 2A–2C). In addition, five of six animals had a marked decrease in platelet counts at the terminal phase of disease (Fig 2D). All animals had evidence of viral RNA in whole blood and plasma by day 6–8 post infection, except for one animal that had evidence of viral RNA in the plasma only at day 10 (Fig 3A and 3B). There was little evidence of viable NiV in either the plasma or whole blood as the virus was undetectable by plaque assay. Previous studies with NiV animal models have only reported viral RNA levels [20, 21, 23, 31–35] so viable NiV may be largely cell associated in the blood. Viral RNA was found in all tissues evaluated with genome copies per mg tissue generally consistent between animals (Fig 3C). The presence of viable virus, as determined by plaque assay, was not as broadly distributed and varied considerably between animals (Fig 3D). Only two animals had evidence of viable virus in the brain despite all animals having viral RNA in the brain at euthanasia. Animals evaluated during these studies were not perfused prior to tissue collection, an approach that may impact the determination of tissue associated virus or viral RNA versus that found in the blood.

### CT and MR imaging

**Fig 4:**
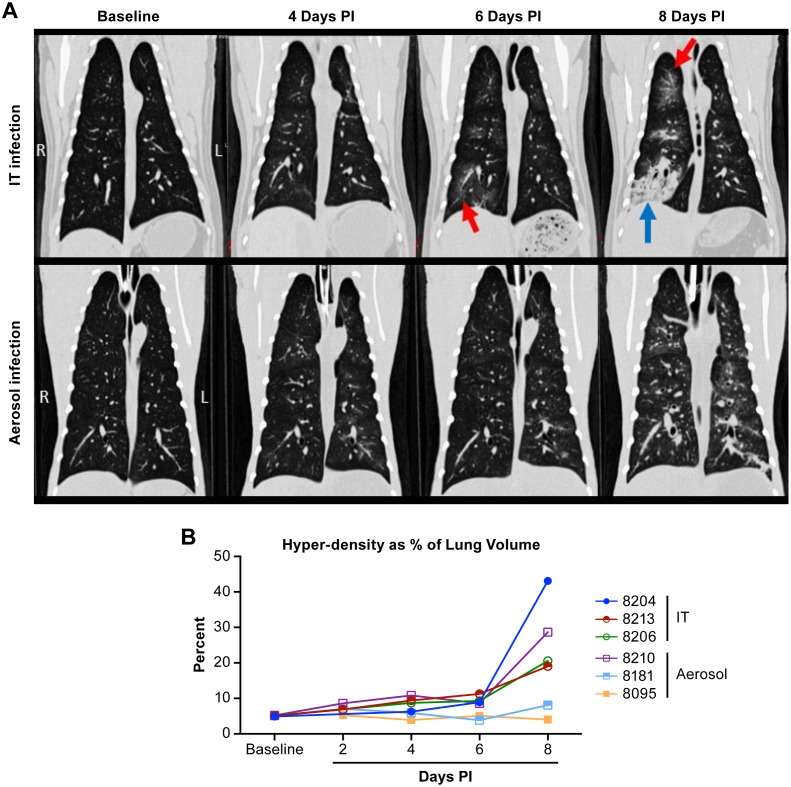
CT analysis of the lungs of NiV infected AGMs. Examples of temporal analysis of changes within the lungs of AGMs infected by intratracheal or aerosol inoculation (A). In animals infected by the IT route, images shows a vascular pattern of infiltration extending to adjacent alveoli, manifested as radiating ground glass opacities (indicated by red arrows), progressing by day 8 to diffuse lobar consolidation (blue arrows) that tends to extend from cranial to caudal in severity. In animals infected by aerosolization of small particles, diffuse progressive thickening of airway walls and adjacent interstitium are typically observable within 6 days of infection, which ultimately extends into the alveoli, uniformly throughout the lungs. Where IT inoculation tends to induce some amount of consolidation, typically in caudal lobes, infection by small particle aerosol appears to cause disseminated pulmonary changes throughout all lobes. Quantification (B) of lung volume demonstrates that animals lost up to 45% of their total lung volume with all three of the animals infected by IT inoculation losing 18% or greater of their lung volume.

Previous studies developing the AGM model for NiV infection utilized IT inoculation to mimic aspirated virus. To understand the potential impact of inhaled virus, we utilized small particle (~1.5 μm) aerosol inoculation in comparison with IT inoculation to determine if there were significant differences in the disease process between these two exposure routes. The use of small particle aerosol for infection was intended to seed the virus deep within the lungs rather than in the bronchial tree as occurs in IT inoculation. In both IT and aerosol exposure groups, CT images indicated minimal changes in the lungs through 4 days after inoculation (Table 1). As disease progressed in animals infected by the IT route, images showed a vascular pattern of infiltration extending to adjacent alveoli, manifested as radiating ground glass opacities with interstitial thickening progressing by day 8 to diffuse lobar consolidation that tended to extend from cranial to caudal in severity (Fig 4A). In animals infected by small particle aerosol, diffuse progressive thickening of airway walls and adjacent interstitium was typically observable within 6 days of infection, ultimately extending into the alveoli, uniformly throughout the lungs (Fig 4A). Where IT inoculation tended to induce some amount of consolidation, typically in caudal lobes, infection by small particle aerosol appeared to cause disseminated vascular and perivascular congestion changes throughout all lobes (Table 1). Quantification of changes in lung volume over the course of disease found that all of the IT inoculated animals had a loss of at least 20% of their lung volume, based on an increase in hyperdense regions quantified in CT images. One animal in the IT inoculation group had a loss of more than 40% of its lung volume (Fig 4B). Only one animal in the aerosol exposure group had a marked decrease in lung volume with a loss of over 25%.

**Table 1:** Radiographic findings in lungs of African green monkeys inoculated with Nipah virus between 2 and 10 days post infection. Imaging and clinical observation were made every two days until the animal met euthanasia criteria.

Animal	Route	Day 2	Day 4	Day 6	Day 8	Day 10
08204	IT	Left dorsal lobe has areas of interstitial thickening that extend into and include the left middle lobe.	Dorsal aspect of left cranial and left caudal lobes show mild thickening of bronchial walls that extends into adjacent alveoli; right middle lobe has minimal focally extensive opacities.	Bilateral changes progressing in severity from cranial to caudal, airway & interstitial thickening radiating into adjacent alveoli.	Diffuse, severe, bilateral consolidation in caudal, right cranial, middle lobes; left cranial lobe spared.	
08213	IT	Lungs fields clear; no changes.	Minimal focal densities right caudal & left middle lobes; Minimal opacities left cranial lobe.	Increasing bilaterally, cranial to caudal infiltrates; cranial lobes hazy opacities radiating into alveoli; caudal lobes; peribronchiolar thickening and central consolidation.	Diffuse vascular congestion in all lobes; increasing in severity from cranial to caudal; right cranial lobe severe consolidation.	
08181	IT	Minimal focal density in dorsal aspect of left middle lobe observed in Baseline. No changes due to disease progression.	Minimal focal density in dorsal aspect of left middle lobe observed in Baseline. No changes due to disease progression.	Cranial lobes clear; right middle & caudal lobes interstitial thickening & minimal adjacent alveolar haziness.	Diffuse pulmonary vascular congestion; increasing central lobular densities with radiating alveolar opacity; moderate severity increasing cranial to caudal.	
08210	Aerosol	Lung fields clear.	Lung fields clear.	Diffuse vascular congestion w/ perivascular edema; mild diffuse haziness throughout lung.	Diffuse interstitial pneumonia w/ diffuse vascular congestion; diffuse parenchymal opacity; caudal and middle lobes more severely affected.	
08095	Aerosol	Minimal thickening of airway walls in dorsal aspect of left cranial lobe; otherwise clear.	Minimal dorsal middle left lobe vascular and bronchial patterns; disseminated.	Mild vascular congestion left caudal lobe; minimal diffuse haziness throughout all lung parenchyma.	Minimal diffuse vascular congestion, most evident in dorsal left caudal lobe; mildly increased haziness adjacent to areas of vascular congestion.	Diffuse vascular congestion, multifocal & coalescing consolidation all lobes; pronounced in left caudal lobe; increased diffuse opacity all lobes.
08206	Aerosol	Misplaced tracheal tube; uninterpretable.	Minimal radiating opacity from central lobar/lobular airways into adjacent alveoli; pronounced right ventral middle lobe.	Moderate diffuse vascular congestion w/ opacity extending into parenchyma of all lung lobes.	Diffuse moderate interstitial pneumonia; diffuse marked vascular congestion; peribronchial edema throughout lung fields.	

**Fig 5:**
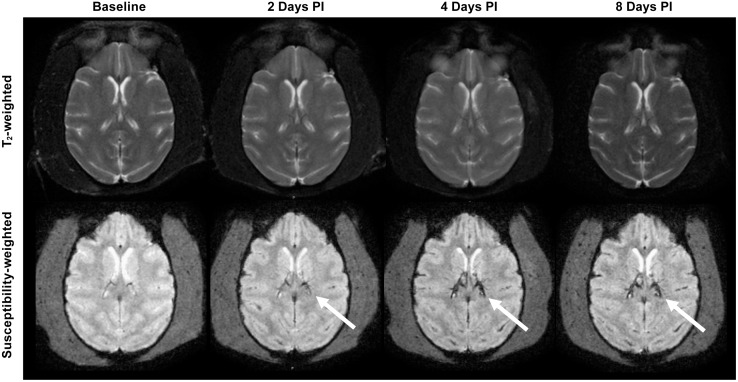
Images of an AGM that underwent MRI before and after aerosol infection with NiV. In this model, neither infection route led to abnormal signal intensity lesions to suggest encephalitis within the brain parenchyma on T2 or on FLAIR weighted images (T2 weighted image, upper row). There was no evidence of abnormal enhancement seen after contrast injection. However, in 2 of the 6 AGMs studied, susceptibility weighted images showed increased visibility/prominence of the deep central and superficial venous structures (white arrows, lower row), compared to baseline, as early as 2 days after infection. This could reflect increased deoxyhemoglobin concentration within the vessels dues to vasodilation and venous stasis possibly part of the systemic manifestations of Nipah virus infection. No parenchymal hemorrhagic changes were seen.

Unlike previously described observations in MR images of acutely ill NiV infected patients [11, 16, 17, 19], neither the IT nor aerosol infection model for NiV induced abnormal signal intensity lesions or changes suggestive of encephalitis within the brain parenchyma. MPRAGE, FLAIR, T_2_-weighted images and T_1_-weighted images before and after contrast injection did not exhibit structural changes or contrast enhancement to suggest disruption of the blood brain barrier over the course of the disease. There was also no clear evidence of meningeal enhancement. However, in 2 of the 6 AGMs studied, susceptibility weighted images showed increased prominence of the deep veins (e.g. internal cerebral, thalamostriate and basal veins) and superficial cortical venous structures as early as 2 days after infection, probably reflecting venous dilation and stasis. However, there were no intraparenchymal hemorrhagic foci (Fig 5). One of these two animals, #8206, also had evidence of viable virus in the brain (Fig 3B). Due to the rapid nature of disease progression in these aerosol and IT exposure models, it is suspected that pulmonary disease progresses too rapidly to allow for brain pathology, such as encephalitis, meningitis or vasculitic ischemic/hemorrhagic changes, to be observed.

### Pathology

**Fig 6:**
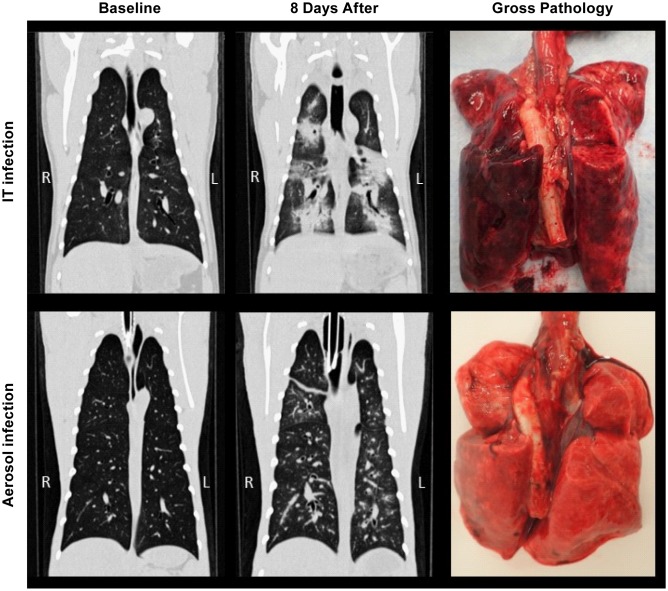
Comparison of CT evaluation of lungs with gross pathology from NiV infected animals. Gross pathologic assessment was in agreement with CT images, indicating severity was often greater in the caudodorsal lung lobes of both models. Extensive lung congestion, hyperemia and multifocal areas of consolidation were more likely to be observed using the IT route of infection, while the aerosol model exhibited mildly congested areas with multifocal areas of hemorrhage. Tracheal mucosa was typically found to be congested, edematous and often hemorrhagic.

Gross pathologic assessment was in agreement with CT images, indicating severity was often greater in the caudodorsal lung lobes of both exposure models (Table 2). Extensive lung congestion, hyperemia and multifocal areas of consolidation were more consistently observed with the IT route of infection, while the aerosol model typically exhibited mildly congested areas with multifocal areas of hemorrhage (Fig 6). In addition, tracheal mucosa was typically found to be congested, edematous and often hemorrhagic and there was evidence of edema in the mediastinum and pericardial connective tissue in all of the animals.

**Table 2:** Gross pathologic assessment at necropsy.

Animal	Route	Lung Lobes	Peripheral Lymph Nodes^1^	Bowel	Spleen	Heart	Other
08204	IT	Extensive congestion, hyperemic, with multifocal areas of consolidation.Tracheal mucosa congested and edematous	Minimally congested & edematous	Diffusely distended with gas; ingesta and digesta minimal	Turgid, slightly friable & engorged with blood	Edema of mediastinum & pericardial connective tissue	Left Kidney enlarged, friable, congested; hemorrhage found in pylorus, duodenum, and urinary bladder mucosa
08213	IT	Mottled, hemorrhagic & congested. Severity greatest in caudodorsal lobes. Tracheal mucosa is hemorrhagic, congested and edematous	Minimally congested & edematous	Diffusely distended with moderate amounts of gas; ingesta and digesta minimal	Turgid, slightly friable	Abundant gelatinous edema of mediastinum and pericardial connective tissue	Urinary bladder and uterus had multifocal mucosal hemorrhage
08181	IT	Slightly congested/hyperemic with a few areas of consolidation. Severity greatest in caudodorsal lobes which have multifocal hemorrhage. Tracheal mucosa is hemorrhagic, congested and edematous	Minimally congested & edematous	Mildly distended with gas; ingesta and digesta minimal	Turgid, slightly friable, congested	Abundant gelatinous edema of mediastinum and pericardial connective tissue	Adrenals slightly congested; pylorus & duodenum have scattered petechial hemorrhages on mucosa, with serosal hemorrhage and edema surrounding the duodenum and pancreas
08210	Aerosol	Mildly congested with small multifocal areas of hemorrhage in cranial and caudodorsal lobes. Tracheal mucosa is congested and edematous	Minimally congested & edematous	Ingesta and digesta minimal	Turgid, slightly friable	Edema of the mediastinum and pericardial connective tissue	Multifocal mucosal hemorrhage of urinary bladder mucosa
08095	Aerosol	Mildly congested with multifocal areas of hemorrhage, primarily in caudodorsal lobes. Tracheal mucosa is congested and edematous	Minimally congested & edematous	Ingesta and digesta minimal	No changes noted	Edema of the mediastinum and pericardial connective tissue	Multifocal mucosal hemorrhage of urinary bladder mucosa
08206	Aerosol	Mildly congested with small multifocal areas of hemorrhage primarily in the caudodorsal lobe	Minimally congested & edematous	Ingest and digesta minimal	Turgid, slightly friable	Edema of mediastinum and pericardial connective tissue	Multifocal mucosal hemorrhage of urinary bladder mucosa

^1^Both axillary and inguinal lymph nodes.

In five of six animals the spleen was noted as turgid and friable while minimal congestion was identified in peripheral lymph nodes (Table 2). Hemorrhage was also noted in the mucosa of the urinary bladder, as has been noted previously [23], and in mucosal surfaces of the intestine in two of the animals exposed by the IT route. All of the animals exposed by the IT route had a significant build-up of gas in the intestines while this was not noted in the aerosol-exposed animals.

Similar to previously reported work, microscopic evaluation of tissue sections identified extensive vasculitis in nearly all tissues examined from each of the animals. Prominent in all of the animals was the presence of syncytia in the spleen, necrohemorrhagic lymphadenitis in the tracheobronchial lymph node, necrohemorrhagic tracheitis and hepatocellular degeneration in the liver. Alterations in the lung observed in CT could be attributed to vasculitis, hemorrhage, edema and bronchointerstitial pneumonia in a number of the animals. None of the animals had evidence of encephalitis, meningitis, ischemia or hemorrhage in the brain. In general, the pathology data gathered from this study suggests a “hemorrhagic” type disease with broadly distributed vasculitis likely the predominant contributor to vascular leakage.

### Systemic immune response to infection

**Fig 7:**
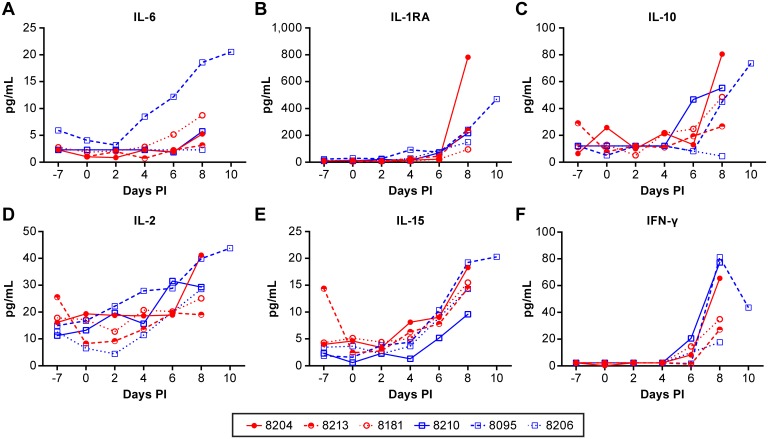
Evaluation of cytokine levels in serum collected from NiV infected AGM over the course of disease. Cytokines associated with inflammation (IL-6, IL-1RA, IL-10) (panels A-C) and Th1 immunity (IL-2, IL-15, IFN-γ) (panels D-F) increase in the serum with disease progression in most animals infected with NiV by IT (black) or aerosol (gray) inoculation. These data demonstrate the presence of a systemic inflammatory response and suggest the onset of a cell-mediated immune response.

In order to understand the immune response to acute NiV infection, plasma cytokine and peripheral blood cell populations were measured over the course of disease. There were minimal changes in most of the cytokines and chemokines measured, however several cytokines were elevated and were indicative of a systemic proinflammatory response with stimulation of cell-mediated immunity. There were no clear differences in the response between the aerosol and IT exposure groups with any of the measured serum cytokine levels. The pro-inflammatory cytokine IL-6 was moderately elevated in several animals in both the aerosol and IT infection groups, but was particularly enhanced in the one animal that had nasal discharge during the aerosol exposure (#8095) (Fig 7). The anti-inflammatory cytokines IL-1RA and IL-10 were also elevated in most of the animals, again with animal #8095 having significantly elevated response with both cytokines (Fig 7). Animal #8204 also had very high IL-1RA and IL10 levels.

Cytokines associated with T cell stimulation became elevated in most of the infected animals as disease progressed, particularly in the late stages of disease (Fig 7). These cytokines included IL-2 and IL-15, which serve biologically related functions associated with development of Th1 immunity. The levels of IFN-γ were also increased in all animals late in the disease process, again supporting the development of a Th1 biased cell-mediated response (Fig 7). Interestingly, animal #8095 was again one of the higher responders, perhaps suggesting an underlying infection that was not apparent prior to study initiation.

**Fig 8:**
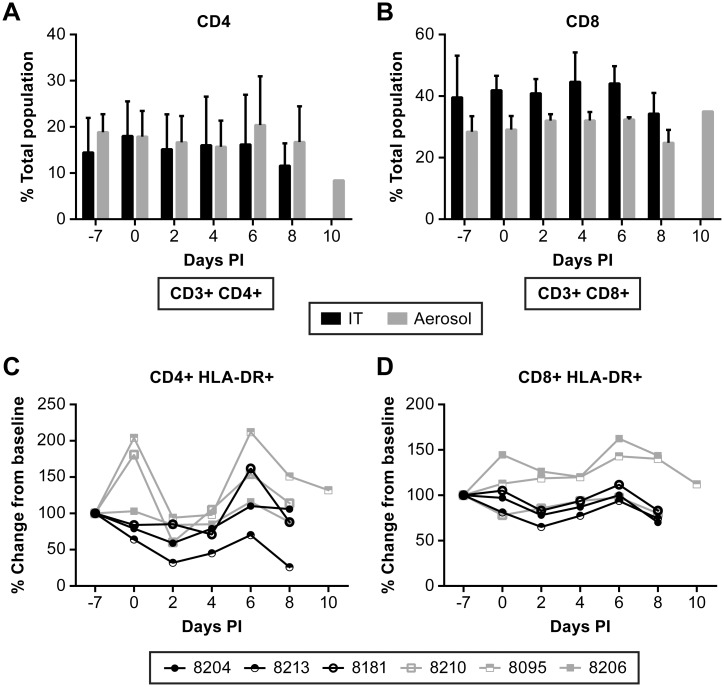
Evaluation of T cell responses in NiV infected AGM over the course of disease. Flow cytometric analysis of changes in CD4+ (A) and CD8+ (B) populations. Evaluation of changes in activated T cell populations was performed using HLA-DR as a marker for activation and was referenced to a pre-infection bleed. No significant changes were noted in activated CD4+ T cells (C), but two animals had nominal increased in activated CD8+ T cells (D) relative to other animals. Animals were infected with NiV by IT (black) or aerosol (gray) inoculation.

Evaluation of peripheral T cell populations found that there was not a significant expansion of either CD4+ or CD8+ T cell populations as might be expected based on cytokine release data (Fig 8A and 8B). However, when CD8+ T cell populations were evaluated for expression of the activation marker HLA-DR, there was a moderate increase in the population of activated CD8+ T cells in two of the aerosol exposed animals, but not in the remaining four animals (Fig 8D). These data, in addition to systemic increases in IL-2 production, suggest expansion of CD8+ effector T cells. These data demonstrate that NiV infection stimulates a Th1-biased cell-mediated response that was detectable beginning about 6 days post-infection. Development of a marked cell-mediated response to NiV infection in this model may take more than the 8–10 days that was the duration of the disease course in this study.

### Tissue specific response to infection

Tissues were collected at necropsy and analyzed for the presence of cytokines, chemokines and for populations of immune cells that could indicate changes in immune status or infiltration of immunoreactive cells. Cytokine and chemokine expression was largely consistent between the IT and aerosol inoculated groups. One animal, #8095, had elevated IL-17A and MIP-1α in the lungs relative to other animals in either the IT or aerosol groups (S6 and S7 Figs). Animal #8095 also had elevated IL-4 levels in the brain compared to other animals in the aerosol group, but that were comparable to animals in the IT inoculation group (S6 and S7 Figs). As previously indicated, this animal survived until 10 dpi, which may have allowed the immune response to progress.

In order to determine if the presence of specific immunoreactive cells could be correlated with disease severity we collected sections of lungs and brain at necropsy from two IT and three aerosol inoculated animals and measured the populations of B cells, T cells, granulocytes, monocytes, macrophages, myeloid dendritic cells (mDC) and natural killer (NK) cells by flow cytometry. Microglia populations were also evaluated in the brain. One of the animals (#8213) in the IT inoculated group succumbed to infection precluding timely collection of viable tissues. As there is little literature available regarding the specific markers on immune cells in AGMs, data from human and rhesus macaque populations were utilized. Markers for cell populations in the lung and, particularly, the brain were based on largely on markers expected for circulating cell populations.

**Fig 9:**
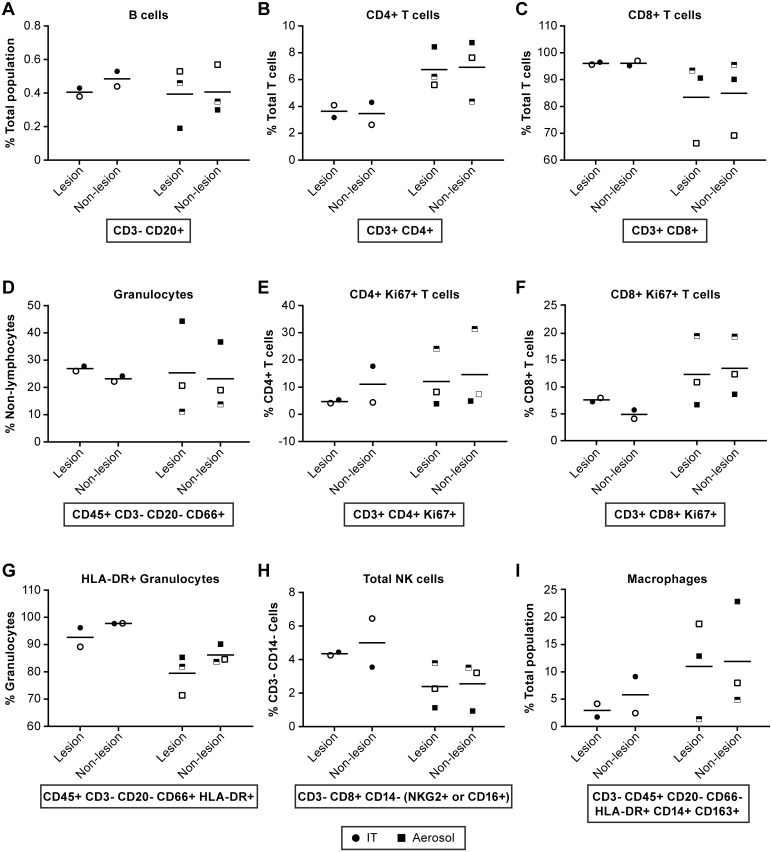
Evaluation of immune cell population in the lung collected at necropsy. Comparisons of immune cell populations in “lesion” and “non-lesion” samples collected at necropsy from animals infected by IT (●) or aerosol (■) inoculation. Markers are consistent for individual animals. IT inoculated: 8204 (●), 8213 (half-filled ○) and 8181 (○); Aerosol inoculated: 8210 (□), 8095 (half-filled □) and 8206 (■). Represented are data for (A) B cells, (B) CD4+ T cells, (C) CD8+ T cells, (D) Granulocytes, (E) Ki67+ CD4+ T cells, (F) Ki67+ CD8+ T cells, (G) HLA-DR+ Granulocytes, (H) Total NK cells and (I) Macrophages. These data suggest that CD4+ cells are elevated in aerosol inoculated animals relative to IT inoculated while activated granulocytes (HLA-DR+) and NK cells appear somewhat elevated in IT inoculated animals. Statistical analyses were not performed due to limited group size.

In the lungs of IT inoculated animals hemorrhagic lesions were readily apparent throughout the tissue (Fig 6). In aerosol inoculated animals, lesions were not visibly apparent so tissues indicated as “lesion” or “non-lesion” should be considered largely equivocal. Evaluation of lymphocyte populations suggested that B cell populations were similar between the IT and aerosol infected groups (Fig 9). A larger percentage of the T cells in the aerosol group were CD4+ T cells compared to the IT group, whereas the IT inoculated group had a larger percentage of CD8+ T cells (Fig 9). Granulocyte populations appeared to be equivocal between the exposure groups. However, in the IT exposure group the granulocyte population had a higher proportion of activated (HLA-DR+) granulocytes (Fig 9). Populations of monocytes, macrophages (Fig 9) and myeloid dendritic cells were similar in both exposure groups. In this study we also found that there were a higher proportion of NK cells in the IT exposure group relative to aerosol exposure and that three distinct populations of NK cells could be differentiated in these animals while using CD16 and NKG2 as markers for NK cells (Fig 9). Animal # 8095, which survived longer than other animals, had elevated numbers of proliferating (Ki67+) CD4+ T cells and NK cells in their lungs relative to other animals. As indicated previously, this animal also had elevated IL-17A and MIP-1α in the lungs relative to other animals (S7 Fig).

**Fig 10:**
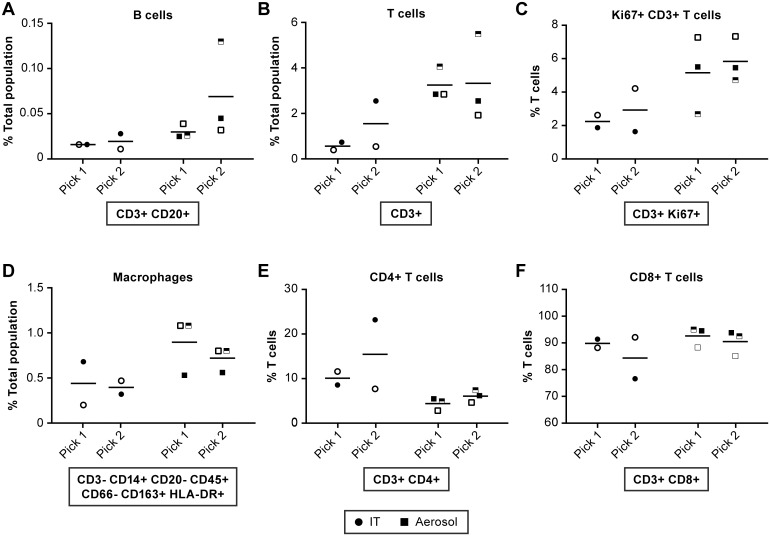
Evaluation of immune cell populations in the brain collected at necropsy. Comparisons of immune cell populations in two samples collected from the cerebral cortex at necropsy from animals infected by IT (●) or aerosol (■) inoculation. Markers are consistent for individual animals. IT inoculated: 8204 (●), 8213 (half-filled ○) and 8181 (○); Aerosol inoculated: 8210 (□), 8095 (half-filled □) and 8206 (■). Represented are data for (A) B cells, (B) T cells, (C) Ki67+ T cells, (D) Macrophages, (E) CD4+ T cells and (F) CD8+ T cells. These data suggest that T cells infiltrated into the brain of aerosol-infected animals and that a higher proportion of these cells are activated. There were also an increased number of macrophages in the brain of two of the animals in the aerosol group. Statistical analyses were not performed due to limited group size.

Lesions were not grossly apparent in the brains of any of the NiV infected AGM. Two samples were collected from the cerebral cortex of all animals, except #8213, for analysis by flow cytometry. There appeared to be an elevation in the total number of CD3+ T cells in the aerosol group compared to the IT inoculated group (Fig 10B) with a correlating proportion of 2–6% Ki67+ indicating cell expansion (Fig 10C). Of the T cells found in the brain, around 90% were CD8+ T cells (Fig 10F). There was a slightly increased number of NK cells and macrophages in the brain of aerosol inoculated animals (Fig 10), but all other cell types evaluated were equivalent between the two groups. Animal #8095 (indicated by a half-filled square) had elevated T cells in the brain relative to other animals (Fig 10B). As indicated previously, this animal also had slightly elevated IL-4 in the brain relative to other aerosol inoculated animals (S7 Fig). IL-4 stimulates B cell differentiation and also T cell proliferation.

## Discussion

In the study described here, the focus was on expanding our understanding of the AGM model for NiV disease by evaluating the impact of small particle aerosol exposure as compared to the previously established IT inoculation model. Disease progression was monitored, in part, by CT and MR imaging to visualize pulmonary and neurological changes without the need for serial sacrifice. In order to gain a broad understanding of the immune response to NiV infection in the AGM model, quantification of cytokine and immune cell populations were measured in blood over the course of the infection and in tissue and blood collected at necropsy.

These studies found that the outcome of NiV infection by the IT or aerosol route was the same, but that disease development was markedly different between the two inoculation routes. In both infection models, severe ARD was the prominent manifestation of disease with no overt signs of neurological involvement, likely due to the rapidly progressing respiratory disease. The use of CT imaging allowed for near real time evaluation of disease progression and for quantification of changes in lung volume over the course of disease. Animals infected by the IT route tended to have a more significant loss of lung volume, based on measurement of hyperdensity within the CT images. The IT inoculated animals also had large focal hyperdensities with edema and consolidation whereas animals exposed by aerosol had more diffuse changes on the CT. While there was evidence of edema in the lungs suggesting a significant local inflammatory immune response, the primary impact of infection in both models was development of systemic vasculitis due to NiV infection and development of virus-induced syncytia, as has been described previously in both the AGM and hamster models for NiV infection [23, 36].

The development of neurological disease has been previously described in humans following NiV infection, particularly during the initial outbreak in Malaysia [7, 15, 37]. Examination of human cases using MRI provided examples of discrete focal lesion within the brain or diffuse cortical involvement [16, 19]. In the studies reported here, there was no overt indication of neurological disease in any of the infected animals. Minimal changes involving venous structures in the brain in two of the animals, as seen by MR imaging, suggested vasodilation and venous stasis, possibly a reflection of systemic vascular compromise. In order to visualize neurological disease in the AGM model, we believe a more protracted disease course is required and that neither the small-particle aerosol nor IT inoculation routes will consistently allow for a longer disease course due to severe pulmonary involvement.

Evaluation of the peripheral immune response through evaluation of changes of cytokines and immune cell populations over the course of infection suggested the initiation of cell-mediated immunity through production of Th1-associated cytokines. However, there were no consistent changes in peripheral CD4+ T cell populations over the course of disease and limited activation of CD8+ T cells in only two animals. The changes in T cell populations correlated somewhat with the Th1 cytokine response in only one animal (#8095); unlike other animals in the study, this animal survived until day 10 and allowed the cell-mediated response to progress. The short eight-day course of disease limited potential development of an adaptive immune response, as would be expected. NiV has been shown to inhibit components of the immune response that would lead to more robust development of adaptive immunity, including interferon signaling and components of the proinflammatory response [38–43]. To date, there is little known about the development of adaptive immunity *in vivo* following NiV infection, clearly demonstrating that further fundamental studies are needed.

An aspect of this study that was particularly challenging was determination of immune cell populations in tissues. Specific markers for atypical immune cells (e.g. NK cells, microglia) are poorly defined in the AGM model making the development of gating strategies in complex flow panels an arduous task. Using the strategy that we developed, we found there were few notable differences between animals inoculated by aerosol or by the IT route, and none could be afforded statistical support given the small number of animals included in this study and inter-animal variation. However, some differences were notable. In particular, the populations of CD4+ and CD8+ T cells in the lung and the blood were comparable. CD4+ T cell populations were high in both the blood and the lung in the IT inoculation group while CD8+ T cells were high in sampled tissues in the aerosol inoculation group. Furthermore, when looking at activation/proliferation markers, activated CD8+ T cells were elevated in the blood and lung of the same two animals in the aerosol group. While there is little statistical strength to these observations, the data suggest that CD8+ T cells are being activated in the periphery and that they are extravasating into the infected lung tissue.

Analysis of granulocyte and NK cells in the lungs suggested increased activation of granulocytes and total populations of NK cells in IT inoculated animals. While not defined in these studies, the granulocytes are most likely neutrophils responding to infected cells and contributing to the congestion seen in the lungs of these animals. Similarly, NK cells may be in higher proportions in the lungs of IT inoculated animals as a component of the large local inflammatory response. The decreased number of granulocytes and NK cells in the lungs of aerosol inoculated animals may reflect the broadly disseminated nature of the lung infection in these animals.

In neither the IT nor the small particle aerosol inoculation model is the neurological model of human disease recapitulated. Anecdotal evidence and published reports have suggested that ARDS is the typical disease seen following NiV infection of humans in Bangladesh, with neurological disease common during the outbreak in Malaysia [1]. Studies in the hamster model have provided unequivocal results with one study suggesting that NiV isolated from Malaysia causes an accelerated infection [34] while another study indicated there was no difference between the two viruses [31]. Recently, Mire et al suggested that a NiV isolated in Bangladesh is more virulent than that isolated in Malaysia [22]. However, the limited size of the Mire et al study makes interpretation of the finding difficult, particularly given the 100% lethality described here at a much lower challenge dose of the Malaysia isolate. It is likely then, that the route of exposure, environmental conditions, medical support or underlying differences in the human populations may make significant contributions to disease development and outcome.

NiV disease has historically been considered “encephalitis” based on indications of neurological disease in Malaysian patients. Given the findings here and in previous work, it appears more appropriate to refer to NiV infection as a “hemorrhagic” type disease in the AGM model given the extent of vasculitis, the hemorrhage seen in the terminal phase of disease, the lack of overt neurological signs and minimal evidence of neurological involvement on MR. In this model, NiV does not appear to be a true neurotropic virus where the brain is the primary target for viral infection, but rather, likely causes neurological manifestations as a result of endothelial barrier breakdown and vascular leakage that allows the virus to access the brain. Extending the course of disease will be critical for understanding the interaction between NiV and the primate brain and for building a model that truly recapitulates the human condition. Further studies to develop a neurological model for NiV infection and to evaluate potential routes of exposure in this model are required before appropriate approaches to therapeutic intervention can be adequately addressed.

In summary, the use of small particle aerosol results in an acute diffuse pulmonary disease of the lower lung that results in a disease course similar to the previously described IT inoculation model. In this study, there was not significant evidence of neurological disease either through cage-side observations, MRI or pathology in either aerosol or IT inoculation groups. The development of overt neurological disease likely requires a more protracted disease course, as would more robust development of a systemic immune response against NiV infection. These studies provide the first evaluation of the host immune response to NiV infection in the AGM model and also demonstrate the value of advanced medical imaging in evaluating and quantifying disease progression without the need for serial sacrifice studies.

## Supporting information

S1 Fig Flow cytometry panels used in this study. Represented are the TNK (A) and Myeloid (C) panels used for tissue staining and the T cell (B) and “Other” cell (D) panel used for staining of PBMCs.
(TIF)

S2 Fig Gating strategy for TNK tissue analysis flow cytometry panel. (TIF)

S3 Fig Gating strategy for peripheral T cell analysis flow cytometry panel. (TIF)

S4 Fig Gating strategy for myeloid tissue analysis flow cytometry panel. (TIF)

S5 Fig Gating strategy for peripheral “other” cell analysis flow cytometry panel. (TIF)

S6 Fig Tissue cytokine response, panel I. Selected cytokine responses in tissues collected from animals inoculated by the intratracheal (A) or aerosol (B) exposure routes.
(TIF)

S7 Fig Tissue cytokine response, panel II. Selected cytokine responses in tissues collected from animals inoculated by the intratracheal (A) or aerosol (B) exposure routes.
(TIF)
