# Supplementary figures and images for: Loss in lung volume and changes in the immune response demonstrate disease progression in African green monkeys infected by small-particle aerosol and intratracheal exposure to Nipah virus
Source: PLoS Negl Trop Dis. 2017 Apr 7;11(4):e0005532. doi: 10.1371/journal.pntd.0005532 (PMC5397074; doi:10.1371/journal.pntd.0005532)

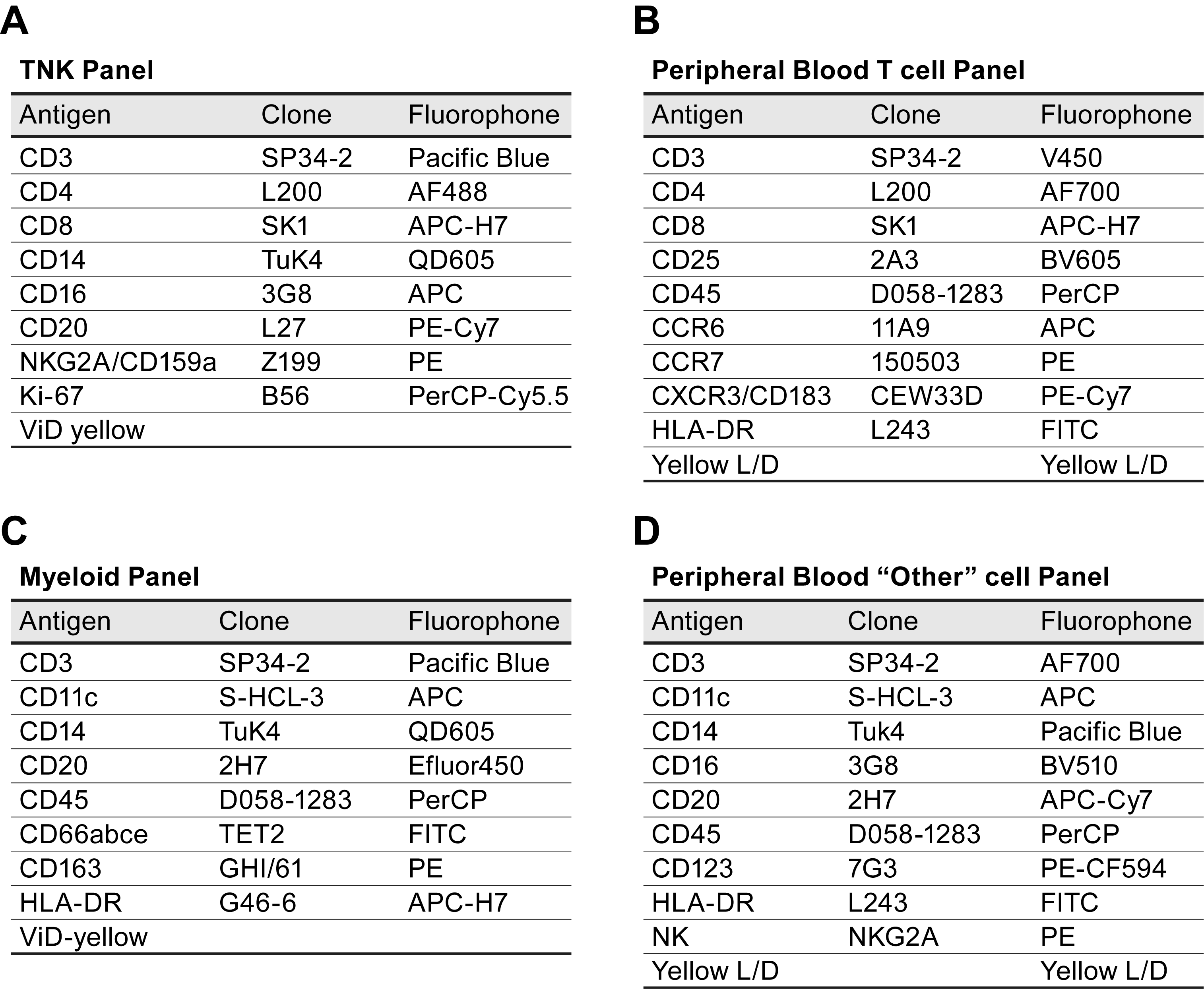

Supplement: S1 Fig — Represented are the TNK (A) and Myeloid (C) panels used for tissue staining and the T cell (B) and “Other” cell (D) panel used for staining of PBMCs. (TIF) [file pntd.0005532.s001.tif]

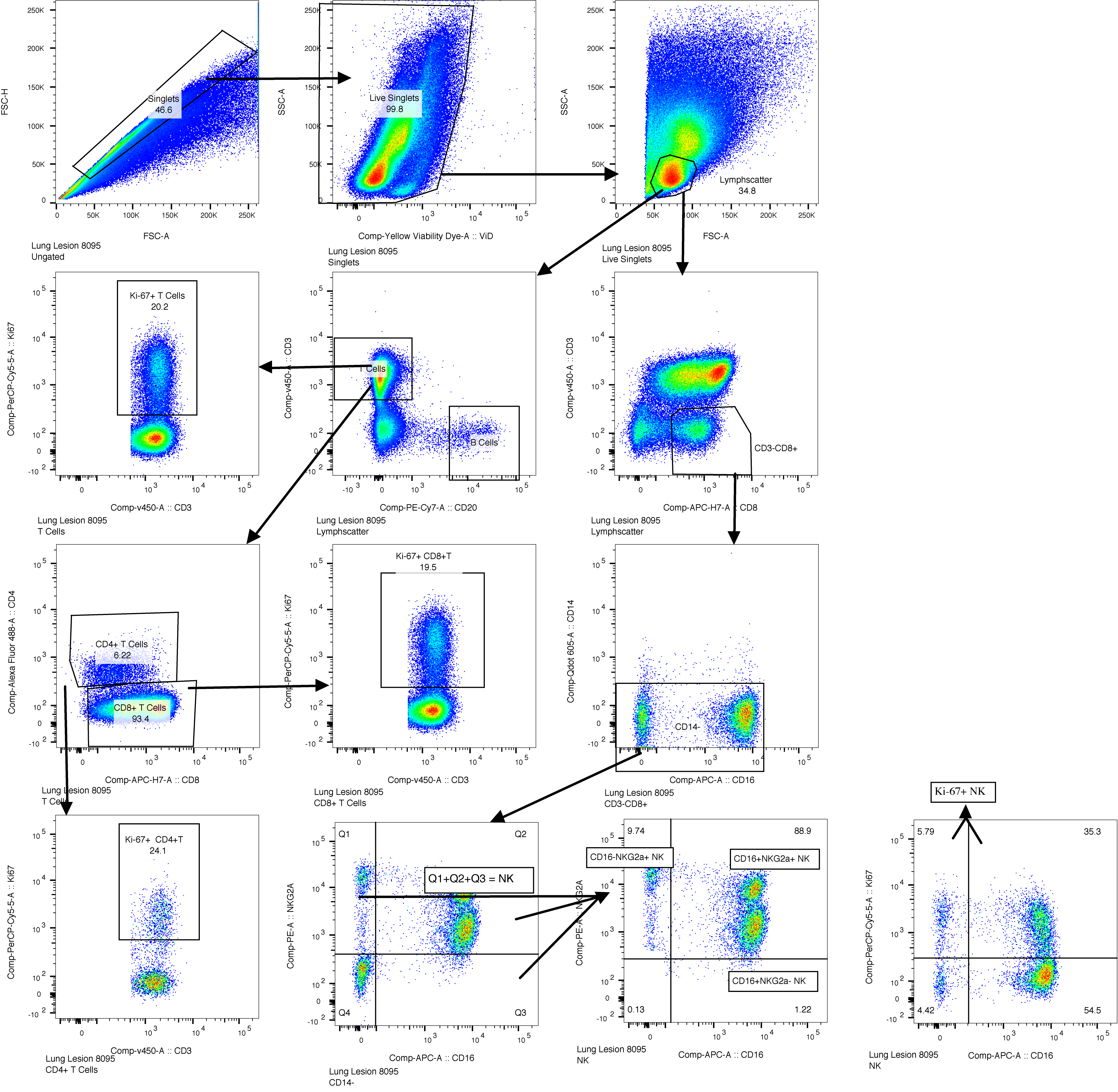

Supplement: S2 Fig — (TIF) [file pntd.0005532.s002.tif]

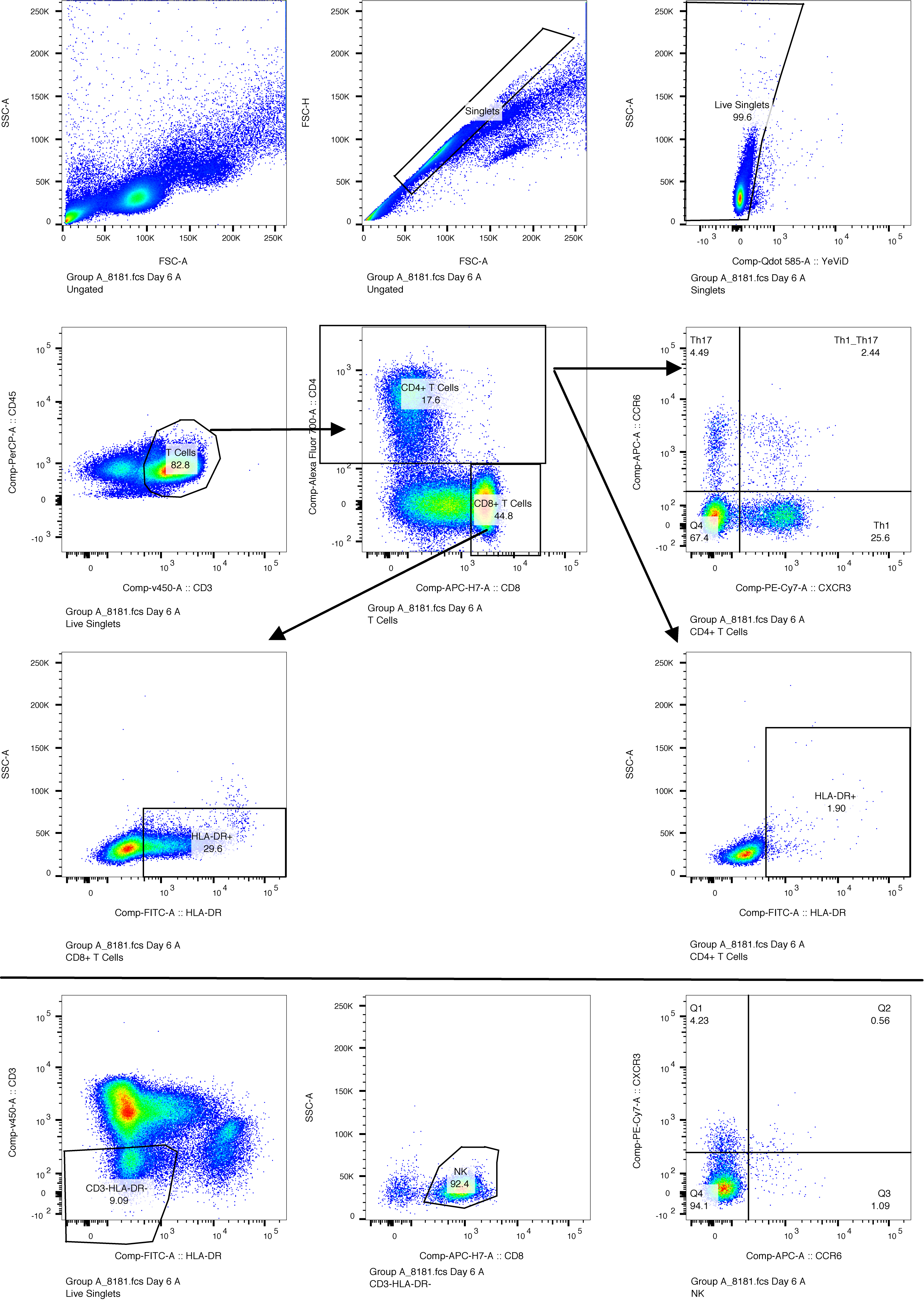

Supplement: S3 Fig — (TIF) [file pntd.0005532.s003.tif]

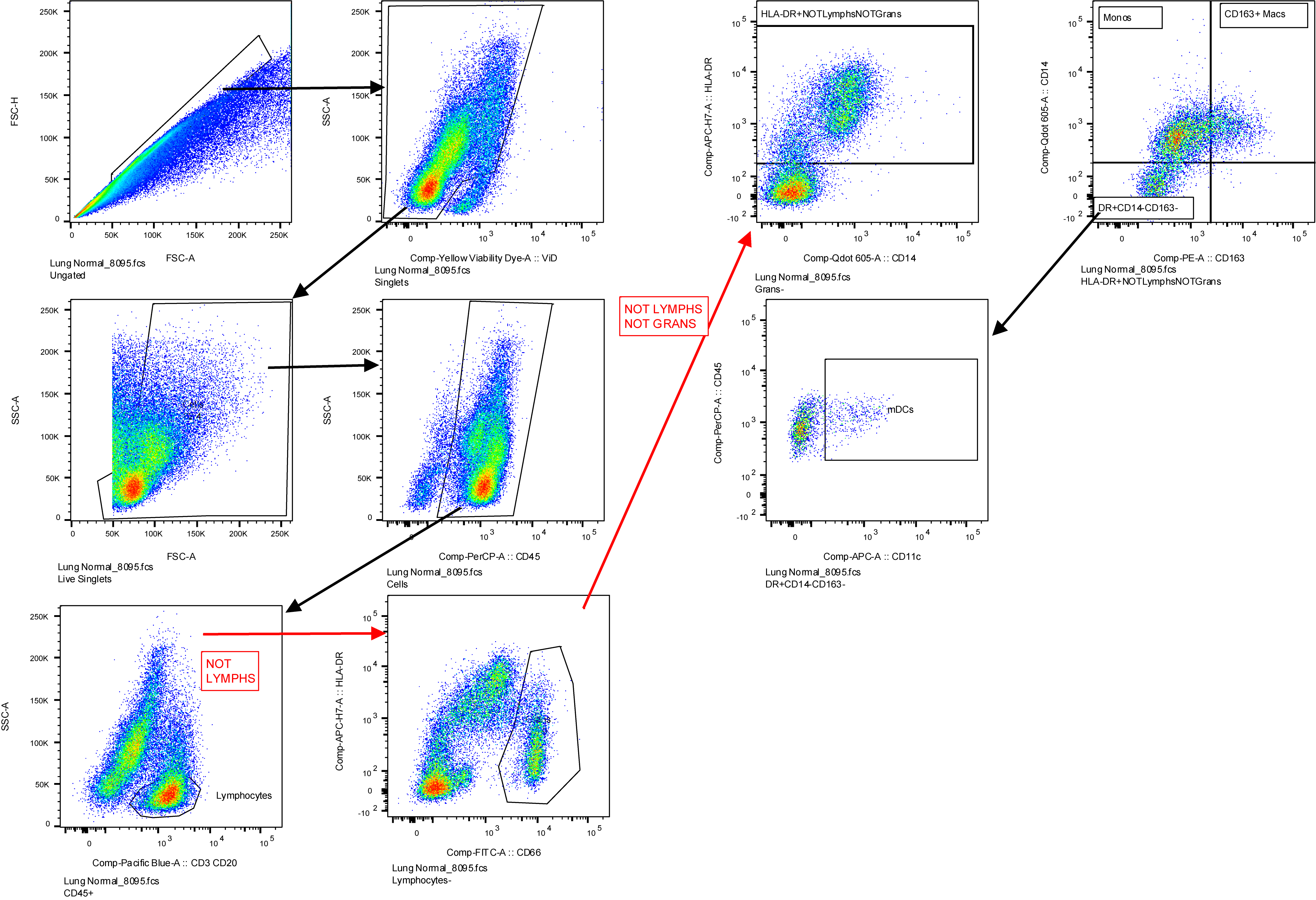

Supplement: S4 Fig — (TIF) [file pntd.0005532.s004.tif]

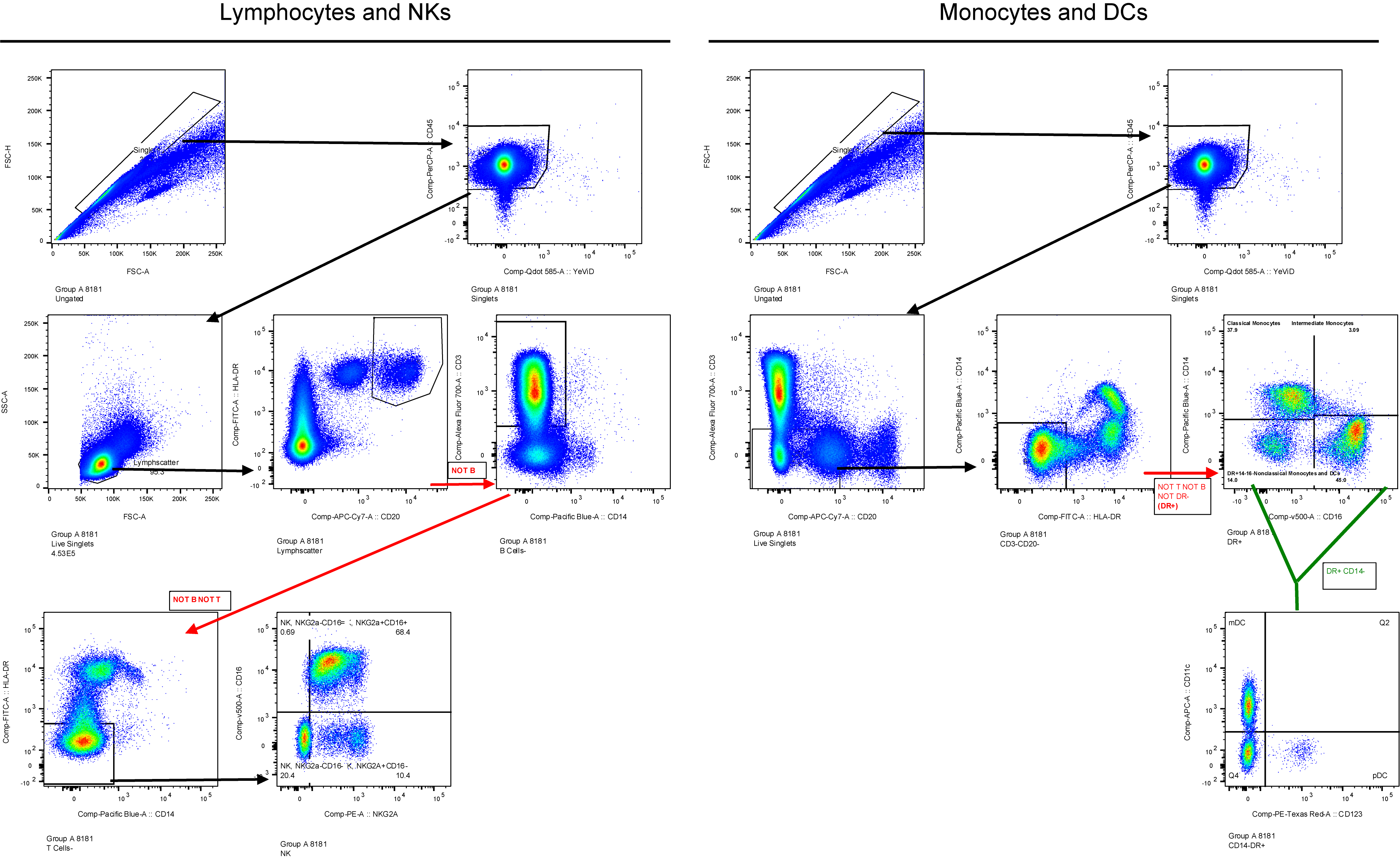

Supplement: S5 Fig — (TIF) [file pntd.0005532.s005.tif]

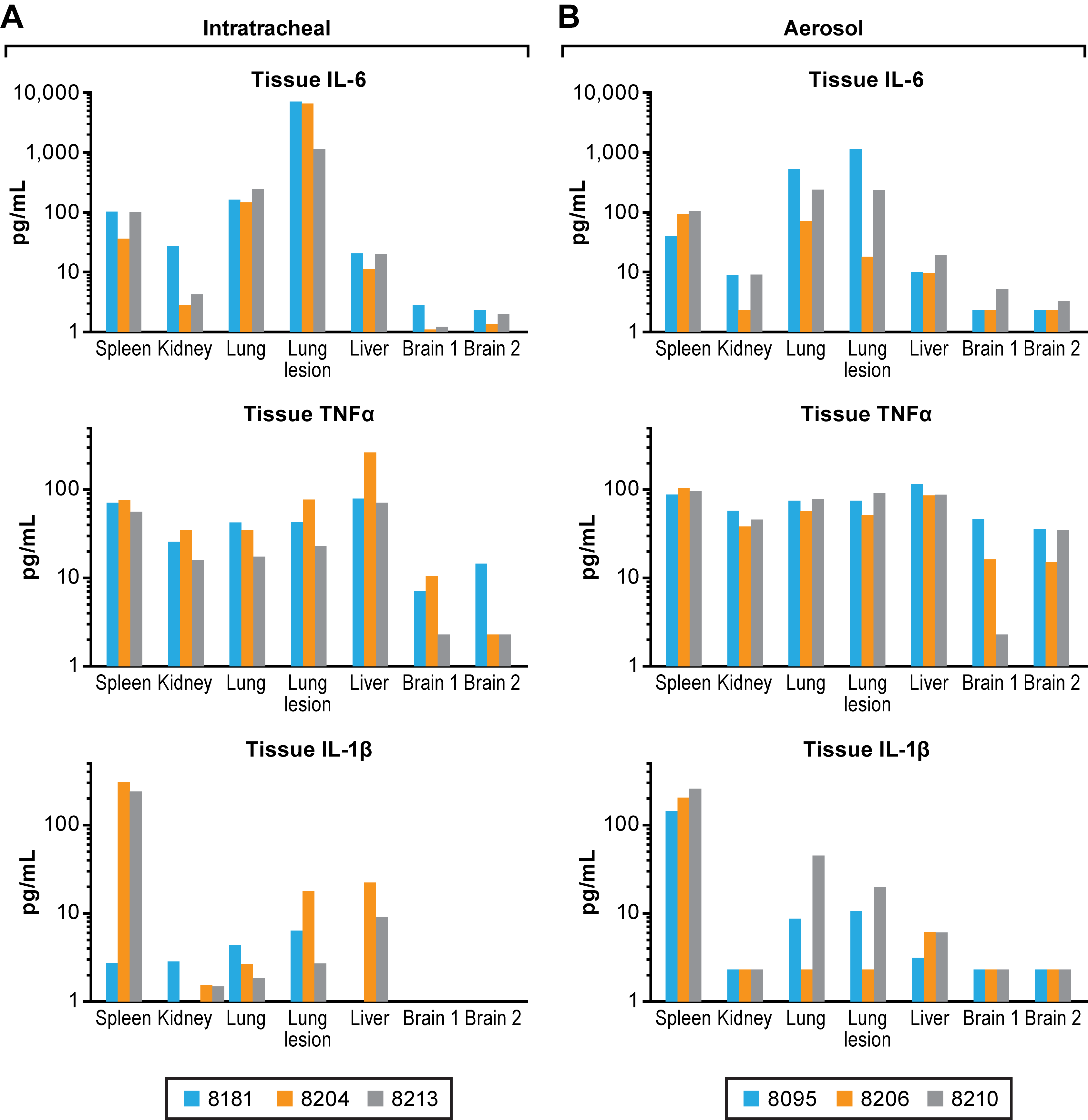

Supplement: S6 Fig — Selected cytokine responses in tissues collected from animals inoculated by the intratracheal (A) or aerosol (B) exposure routes. (TIF) [file pntd.0005532.s006.tif]

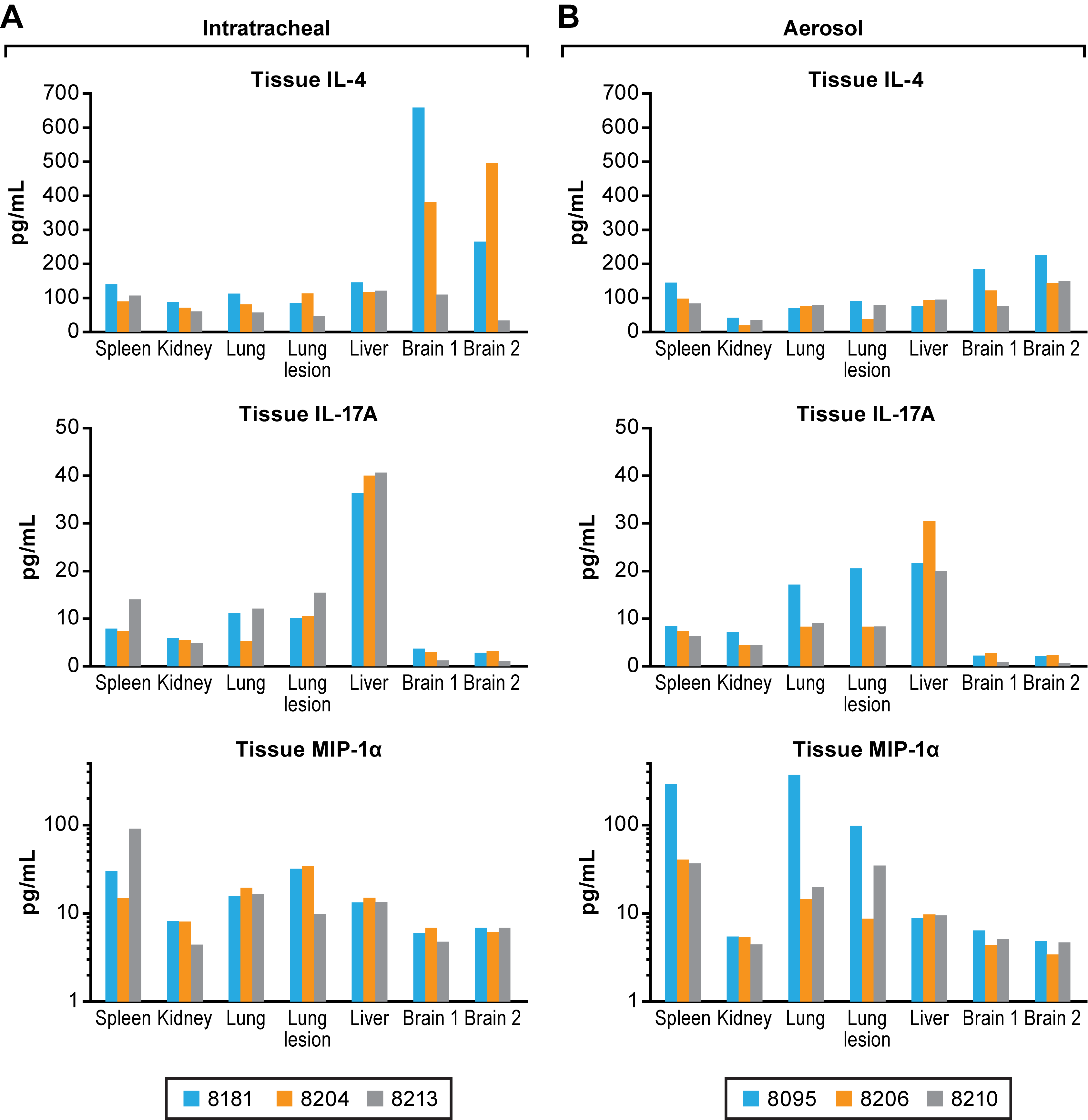

Supplement: S7 Fig — Selected cytokine responses in tissues collected from animals inoculated by the intratracheal (A) or aerosol (B) exposure routes. (TIF) [file pntd.0005532.s007.tif]
